# Nanomaterials for biomedical applications

**DOI:** 10.3762/bjnano.16.105

**Published:** 2025-08-28

**Authors:** Iqra Zainab, Zohra Naseem, Syeda Rubab Batool, Filippo Pierini, Seda Kizilel, Muhammad Anwaar Nazeer

**Affiliations:** 1 Biomaterials and Tissue Engineering Research Laboratory, National Textile University, Faisalabad 37610, Pakistan; 2 School of Engineering and Technology, National Textile University, 37610, Faisalabad, Pakistan; 3 Department of Biosystems and Soft Matter, Institute of Fundamental Technological Research, Polish Academy of Sciences, Warsaw, Poland; 4 Chemical and Biological Engineering, Koç University, Sariyer 34450, Istanbul, Turkey; 5 Research Center for Translational Medicine, Koç University, Sariyer 34450, Istanbul, Turkey

**Keywords:** biomedical applications, drug delivery, nanocarriers, nanomaterials, nanomedicine, nanoparticles, polymeric nanoparticles, tissue regeneration

Medicine has rapidly advanced over the last few decades, and nanotechnology has played a significant role in this progress. The use of nanomaterials has notably contributed to advancements in the fields of disease diagnosis, treatment, and prevention. They can closely interact with cells and molecules of the body due to their small size, which helps to diagnose, enhance imaging, and repair damaged tissues. Nanomaterials have gained popularity in medicine because they can be altered according to the need [1]. Researchers can tailor the shape, surface chemistry, and other specific properties of materials to deliver desirable traits. In particular, some nanoparticles can be used to deliver drugs to a tumor, reducing adverse effects and increasing the success rate of the delivery. Since nanomaterials can be tunable, the vast majority of health sectors are investigating their potential in a wide range of applications, such as targeted drug delivery, gene therapy, tissue regeneration, imaging, and diagnostic tools [2].

Polymeric nanoparticles, dendrimers, and carbon-based structures such as carbon nanotubes are very promising when it comes to delivering drugs and genes to certain parts of the body [3]. Conversely, metallic nanoparticles such as gold and iron oxide, and fluorescent quantum dots have applications in diagnostics and medical imaging. Using these materials, physicians diagnose diseases earlier and more accurately than before [4]. In tissue engineering, nanofibers are being used to develop scaffolds to promote the proliferation of cells. These scaffolds aid patients suffering from chronic wounds as they promote the healing of damaged tissues or organs [5]. Photothermal treatments are also possible with nanomaterials, as specially prepared particles can heat up and destroy cancer cells when exposed to light [6]. Moreover, nanomaterials can be incorporated into implants and prosthetics for enhanced strength, more effective functioning, and biological compatibility [7]. Nanomaterials play a leading role in drug delivery and gene transportation. Older methods for drug delivery usually do work well, miss their intended targets, and cause unwanted side effects. Nanotechnology helps with this challenge by designing nanomaterials that can bring the drug to the required place and then release it in a planned way. One of the earliest nanocarriers examined for drug delivery was liposomes. They are tiny spheres made of lipids, either naturally or synthetically, and their structure is closely similar to the cell membranes [8]. They can carry both water-soluble and fat-soluble drugs, shielding them from breaking down and extending their circulation time. Certain liposome-based drugs have already been approved for therapeutic use, especially in cancer therapy, where they may protect nearby healthy cells from toxicity [9]. Along with liposomes, polymeric nanoparticles have turned out to be an equally dynamic platform. Typically, these particles are composed of biodegradable materials, such as poly(lactic-*co*-glycolic acid) (PLGA) or chitosan. One of the main advantages of polymers is that they can be designed to react to certain bodily conditions, for example, changes in pH or enzymes. As a result, drugs only target affected parts of the body, lowering the damage to healthy cells. Additionally, the surface of these nanoparticles can be modified by attaching antibodies or ligands, which allows these nanoparticles to target particular cell types, making them precise [10]. Dendrimers are considered a more promising group among nanocarriers. They are highly branched structures, with a given shape and many functional groups placed on their surface. This enables the safe loading of high quantities of drugs and their controlled release. They are also being investigated as gene delivery agents since they can transport DNA or RNA, making them a potential candidate for therapies such as gene therapy and RNA-based vaccines [11]. Furthermore, carbon nanotubes have revealed promising results in targeted delivery. Drugs or genetic material can be carried by these cylindrical nanoparticles and directed towards specific cells through external stimuli such as a magnetic field or light [12]. A new nanoscale drug delivery system has been developed by using carbon nanotubes and a carbon nanotube–graphene hybrid to more effectively treat brain cancer cells. These nanocarriers helped drugs stay longer in the body, get to the brain tumor by crossing protective barriers, and directly target cancer cells [13]. However, carbon nanotubes require further investigation before being implanted into the human body due to their toxicity and biocompatibility [14]. In general, smart drug delivery systems developed from nanomaterials are changing the concept of delivering therapies. With lower drug dosing, it helps patients quickly recover with fewer side effects, which is necessary for chronic or life-threatening diseases. Due to all of these advantages, these technologies will become increasingly prevalent in clinical practices [15].

Besides improving treatments, nanotechnology has also advanced the process of monitoring and diagnosing diseases. Being diagnosed early and accurately significantly helps patients with serious conditions such as cancer, neurological disorders, and infections. Quantum dots are a widely studied type of nanomaterial used in biomedical diagnostics. When these semiconductor particles are exposed to light, they strongly and stably emit fluorescence. Unlike regular dyes, quantum dots do not quickly fade and can be adjusted to emit different colors based on their size. This reason makes them ideal for simultaneously spotting and examining various biological environments, which aids both laboratory and clinical research [16]. Gold nanoparticles are also an essential group in this field. Optical properties of these particles are highly dependent on their size and shape. In a diagnostic test, these nanoparticles remarkably improve imaging contrast and even create noticeable color changes, which is useful in biosensors [17]. Gold nanoparticles were being used in certain COVID-19 test kits that change color upon detecting the virus. Their capability to attach to specific antibodies or DNA strands makes them perfect for detecting even faint traces of disease [18]. Moreover, magnetic nanoparticles specially made from iron oxide are also being used in medical imaging. They are usually employed as contrast agents in magnetic resonance imaging (MRI) process [19]. There is more clarity in MRI images due to these nanoparticles, which help doctors to see even the smallest details in tissues and organs [20]. Apart from imaging, magnetic nanoparticles are now used in biosensors and lab-on-a-chip devices for quick and accurate identification of biomarkers from blood or similar samples [21]. A novel biosensor type, called a biosensing drug delivery system, combines sensors with drug release mechanisms. This could make it easier to treat chronic diseases such as diabetes, heart disease, and cancer. When blood sugar and cholesterol levels change within the body, these systems can detect and automatically release the right amount of the medicine that is needed [22]. Point-of-care diagnostics have become possible with these nanomaterials, which allow fast and correct testing results even outside laboratories. Such tools are most helpful in situations where getting advanced diagnostic equipment on site is not possible. Knowing about a disease early allows for selecting the right treatment, which may benefit patients and save lives. Yet, there are a few struggles that need attention. With the expansion of their production, these materials have to be stable and safe in the body, and they must comply with regulatory requirements before healthcare sectors can rely on them. So far, the achievements in nanotechnology for diagnostics seem positive, and we can expect this area to keep improving as additional studies and trials are carried out [23]. The connection between regenerative medicine and tissue engineering applications is notable, as regenerative medicine aims to repair damaged tissues and organs, and nanotechnology is driving major progress in this field [24]. Usually, traditional care works to ease symptoms or slow the disease, but through tissue engineering, the aim is to restore full functioning by supporting the healing capability of the body. Nanomaterials play an important role in this process as they better mimic the natural environment of cells than conventional materials. In tissue engineering, scaffolds are the main element that assists cells to grow, organize, and create new tissue [25]. Nanofibers are ideal in creating scaffolds because they have a large surface area and resemble the natural extracellular matrix (ECM). Moreover, nanofibers offer a proper place for cells to spread, stay attached, and turn into different cell types. Electrospun polymers could produce these nanofibers, which are widely applied for nerve treatment, bone growth, and wound healing [26]. A second approach is the utilization of nano-patterned surfaces. These surfaces have carefully designed nanoscale features, which can be used to control the way the cells function. Surface patterns, for example, can tell cells when to divide, which way to grow, or what type of tissue they should make. It proves to be valuable in stem cell therapy as well as in the development of artificial organs [27].

In recent years, the scientific community has started focusing on what nanocomposites potentially offer. Binding nanoparticles along with biomaterials enhances their strength, flexibility, and durability. For example, adding hydroxyapatite nanoparticles to a polymer can improve bone compatibility, making it well-suited for orthopedic implants. Likewise, the addition of silver nanoparticles provides antibacterial properties for the material. This action is helping to prevent infection during the recovery time [28–29]. One of the major advantages of using nanomaterials in tissue engineering is that they can interact directly with cells at the molecular level [30]. This enables scientists to control not only the physical structure of the tissue but also the biological signals that control cell behavior. Releasing growth factors in a controlled way can be a feature of nanostructured scaffolds to support tissue growth over time. Although the potential is huge, it is still a challenge for researchers to ensure that engineered tissues remain safe, generate the proper immune responses, or are well-integrated into living bodies. However, the advancements so far demonstrate that nanomaterials may be used in the future for tissue recovery, which was previously thought to be beyond repair [31]. In addition to diagnosis and tissue regeneration, nanomaterials can create new treatment methods and enhance the design and performance of medical implants. An area that is getting a lot of attention is photothermal therapy (PTT), in which heating special nanoparticles with light helps kill damaged cells, most notably cancerous cells. Most doctors are using this technique since it treats tumors more precisely and in a less invasive way than standard chemotherapy and radiation [32]. Gold and silver metallic nanoparticles are most often used in photothermal therapy. These nanomaterials can capture specific wavelengths of light from the near-infrared spectrum and then use that energy to generate heat. This heat from the laser points causes cancer cells to be damaged or destroyed, while reducing the damage to nearby healthy tissues [33]. Scientists are also investigating carbon-based nanomaterials such as graphene and carbon nanotubes for different possible uses. Besides their great photothermal properties, these materials can be modified to specifically target tumor cells [34]. Additionally, photothermal nanomaterials are emerging as revolutionary tools in ophthalmology, offering targeted and minimally invasive therapies for conditions such as glaucoma, ocular tumors, and lens opacities. In addition to improving drug delivery and imaging in the eye, these materials have the potential to outperform existing treatments. The research is still in its early stages, and the results are encouraging so far. In the future, machine learning, biocompatible materials, and tailored therapies will all be included for patient-specific care [35].

Nanomaterials are also widely used in medical devices and prosthetics. Traditional implants face various problems, such as poor biocompatibility, inflammatory response, or the risk of infection. However, with the integration of nanomaterials, these devices show higher biocompatibility, durability, and defenses against bacteria [36]. For example, incorporating titanium dioxide or silver nanoparticles in joint replacements or dental implants can decrease the chances of infections and support healing [37]. On implants, nanoscale coatings are also being used to mimic natural tissues more closely and to avoid immune rejection from the body. It is possible to make materials that slowly release therapeutic compounds over time. For instance, anti-inflammatory medications or antibiotics benefit the patient over a longer period without additional procedures. Bone-related implants have demonstrated that nanoscale surfaces stimulate the growth and attachment of bone cells, allowing for faster and safer integration [38]. The key reason nanomaterials are suitable for these applications is that they can function at the cellular and molecular level. This implies that implants with nanomaterials are not only physically compatible with the body but also contribute to biological processes [39]. Therefore, patients can benefit from faster healing, durable implants, and fewer challenges after surgery. Studies are still being conducted to ensure their long-term safety, so their use for therapeutics and tissue structure continue to expand. From cancer therapies to advanced prosthetics, nanomaterials are crucial for developing the latest generation of medical technologies that are smart and responsive to the body natural system [40].

In conclusion, the biomedical field now relies on nanotechnology, which leads to helpful, practical answers when traditional approaches do not work. It turns out that nanomaterials have many uses in medicine, from targeted drug delivery to improved imaging, promoting tissue regeneration, and enhancing the functions of implants and prosthetic devices. Their adaptability makes them so powerful as scientists can change the structure, chemistry, or properties of a material to meet specific requirements, whether to attack cancer cells, allow tissue regrowth, or eliminate bacteria from a surgical site. Since nanoparticles are extremely small, they have the potential to interact with cells and enable cellular events. Even though these technologies are intricate, their usage in everyday healthcare is becoming more evident. Liposomes and polymeric nanoparticles are already being used in certain cancer and vaccine treatments [41]. Detection of diseases at an early stage is becoming more possible with gold nanoparticles or quantum dots. Scaffolds made from nanofibers and implants with coatings are about to become more common in surgeries and regenerative therapies. However, it is necessary to consider issues related to long-term safety, mass production, and the way these materials interact with the body. As technology continues to advance, regulatory standards should also be developed so that patients can access innovations safely. For these materials to be successfully used, it will be important for scientists, doctors, and regulators to communicate openly and gain the trust of the public. Nanomaterials may prove to be highly important in shaping the future of medicine. They not only enhance what we already have, but also help to develop new types of treatments and diagnostics with more effectiveness. Ongoing research, responsive development, and collaboration might enable nanotechnology to transform modern healthcare on an unprecedented scale [42].

Iqra Zainab, Zohra Naseem, Syeda Rubab Batool, Filippo Pierini, Seda Kizilel and Muhammad Anwaar Nazeer

Faisalabad, Warsaw, and Istanbul, August 2025

## Data Availability

Data sharing is not applicable as no new data was generated or analyzed in this study.

## References

[1] Joseph T, Kar Mahapatra D, Esmaeili A, Piszczyk Ł, Hasanin M, Kattali M, Haponiuk J, Thomas S (2023). Nanomaterials.

[2] Naseem Z, Zainab I, Batool S R, Nazeer M A, Shaker K, Nawab Y (2025). Biomedical Materials. Engineering Materials: Fundamentals, Processing and Properties.

[3] Zare H, Ahmadi S, Ghasemi A, Ghanbari M, Rabiee N, Bagherzadeh M, Karimi M, Webster T J, Hamblin M R, Mostafavi E (2021). Int J Nanomed.

[4] Tufani A, Qureshi A, Niazi J H (2021). Mater Sci Eng, C.

[5] Rybak D, Du J, Nakielski P, Rinoldi C, Kosik‐Kozioł A, Zakrzewska A, Wu H, Li J, Li X, Yu Y (2025). Adv Healthcare Mater.

[6] Pierini F, Nakielski P, Urbanek O, Pawłowska S, Lanzi M, De Sio L, Kowalewski T A (2018). Biomacromolecules.

[7] Gautam S, Bhatnagar D, Bansal D, Batra H, Goyal N (2022). Biomed Eng Adv.

[8] Fulton M D, Najahi-Missaoui W (2023). Int J Mol Sci.

[9] Benderski K, Lammers T, Sofias A M (2025). Nat Nanotechnol.

[10] Elmowafy M, Shalaby K, Elkomy M H, Alsaidan O A, Gomaa H A M, Abdelgawad M A, Mostafa E M (2023). Polymers (Basel, Switz).

[11] Kisakova L A, Apartsin E K, Nizolenko L F, Karpenko L I (2023). Pharmaceutics.

[12] Rahamathulla M, Bhosale R R, Osmani R A M, Mahima K C, Johnson A P, Hani U, Ghazwani M, Begum M Y, Alshehri S, Ghoneim M M (2021). Materials.

[13] Milenkovska R, Geskovski N, Shalabalija D, Mihailova L, Makreski P, Lukarski D, Stojkovski I, Simonoska Crcarevska M, Mladenovska K (2025). Beilstein J Nanotechnol.

[14] Zhang W, Zhang Z, Zhang Y (2011). Nanoscale Res Lett.

[15] Patra J K, Das G, Fraceto L F, Campos E V R, Rodriguez-Torres M d P, Acosta-Torres L S, Diaz-Torres L A, Grillo R, Swamy M K, Sharma S (2018). J Nanobiotechnol.

[16] Volkov Y (2015). Biochem Biophys Res Commun.

[17] Kondorskiy A D, Lebedev V S (2021). J Russ Laser Res.

[18] Ardekani L S, Thulstrup P W (2022). Nanomaterials.

[19] Demirer G S, Okur A C, Kizilel S (2015). J Mater Chem B.

[20] Rümenapp C, Gleich B, Haase A (2012). Pharm Res.

[21] Jamshaid T, Neto E T T, Eissa M M, Zine N, Kunita M H, El-Salhi A E, Elaissari A (2016). TrAC, Trends Anal Chem.

[22] Akolpoglu M B, Bozuyuk U, Erkoc P, Kizilel S, Inamuddin K R, Mohammad A, Asiri A M (2019). Biosensing–Drug Delivery Systems for In Vivo Applications. Advanced Biosensors for Health Care Application.

[23] Joyce P, Allen C J, Alonso M J, Ashford M, Bradbury M S, Germain M, Kavallaris M, Langer R, Lammers T, Peracchia M T (2024). Nat Nanotechnol.

[24] Alzate-Correa D, Lawrence W R, Salazar-Puerta A, Higuita-Castro N, Gallego-Perez D (2022). AAPS J.

[25] Nazeer M A, Yilgor E, Yilgor I (2019). Polymer.

[26] Zainab I, Naseem Z, Batool S R, Waqas M, Nazir A, Nazeer M A (2025). Beilstein J Nanotechnol.

[27] Handrea-Dragan I M, Botiz I, Tatar A-S, Boca S (2022). Colloids Surf, B.

[28] George S M, Nayak C, Singh I, Balani K (2022). ACS Biomater Sci Eng.

[29] Kaabipour S, Hemmati S (2021). Beilstein J Nanotechnol.

[30] Turon P, Del Valle L, Alemán C, Puiggalí J (2017). Appl Sci.

[31] Zhang L, Webster T J (2009). Nano Today.

[32] Deng X, Shao Z, Zhao Y (2021). Adv Sci.

[33] Yamada M, Foote M, Prow T W (2015). Wiley Interdiscip Rev: Nanomed Nanobiotechnol.

[34] Sajjadi M, Nasrollahzadeh M, Jaleh B, Soufi G J, Iravani S (2021). J Drug Targeting.

[35] Zhuang J, Jia L, Li C, Yang R, Wang J, Wang W-a, Zhou H, Luo X (2025). Beilstein J Nanotechnol.

[36] Yang W, Ratinac K R, Ringer S P, Thordarson P, Gooding J J, Braet F (2010). Angew Chem, Int Ed.

[37] Gallo J, Panacek A, Prucek R, Kriegova E, Hradilova S, Hobza M, Holinka M (2016). Materials.

[38] Stich T, Alagboso F, Křenek T, Kovářík T, Alt V, Docheva D (2022). Bioeng Transl Med.

[39] Liu Z, Liu X, Ramakrishna S (2021). Introduction and Overview. An Introduction to Circular Economy.

[40] Nadtoka O, Virych P, Krysa V, Chumachenko V, Kutsevol N, Fesenko O, Yatsenko L (2023). Hydrogel Materials for Biomedical Application: A Review. Nanooptics and Photonics, Nanochemistry and Nanobiotechnology, and Their Applications. Springer Proceedings in Physics, vol 280.

[41] Haleem A, Javaid M, Singh R P, Rab S, Suman R (2023). Global Health J.

[42] Hasan A, Morshed M, Memic A, Hassan S, Webster T J, Marei H E-S (2018). Int J Nanomed.

